# Roles of PDE1 in Pathological Cardiac Remodeling and Dysfunction

**DOI:** 10.3390/jcdd5020022

**Published:** 2018-04-23

**Authors:** Si Chen, Walter E. Knight, Chen Yan

**Affiliations:** 1 Aab Cardiovascular Research Institute, Department of Medicine, University of Rochester School of Medicine and Dentistry, Rochester, NY 14641, USA; si_chen@urmc.rochester.edu; 2 Department of Pharmacology and Physiology, University of Rochester School of Medicine and Dentistry, Rochester, NY 14641, USA; 3 Division of Cardiology, Department of Medicine, University of Colorado School of Medicine, 12700 E. 19th Avenue, B139, Aurora, CO 80045, USA; wek4@cornell.edu

**Keywords:** phosphodiesterases (PDEs), PDE1, cyclic nucleotide, cardiac hypertrophy, cardiac dysfunction

## Abstract

Pathological cardiac hypertrophy and dysfunction is a response to various stress stimuli and can result in reduced cardiac output and heart failure. Cyclic nucleotide signaling regulates several cardiac functions including contractility, remodeling, and fibrosis. Cyclic nucleotide phosphodiesterases (PDEs), by catalyzing the hydrolysis of cyclic nucleotides, are critical in the homeostasis of intracellular cyclic nucleotide signaling and hold great therapeutic potential as drug targets. Recent studies have revealed that the inhibition of the PDE family member PDE1 plays a protective role in pathological cardiac remodeling and dysfunction by the modulation of distinct cyclic nucleotide signaling pathways. This review summarizes recent key findings regarding the roles of PDE1 in the cardiac system that can lead to a better understanding of its therapeutic potential.

## 1. Introduction

Heart failure, the inability of the heart to provide sufficient blood to the body, is a leading cause of death in the United States. Heart failure is associated with significant myocardial deterioration, including pathological hypertrophy, fibrosis, and cell death, as well as contractile dysfunction and ventricular arrhythmia [1]. Therefore, identifying novel molecular targets involved in pathological cardiac remodeling and dysfunction is crucial. Cyclic nucleotide signaling is important in numerous biological functions and pathological processes in the cardiovascular system, ranging from short-term muscle contraction/relaxation to long-term cell growth/survival and structural remodeling [2]. Phosphodiesterases (PDEs), by catalyzing the hydrolysis of cyclic nucleotides, play important roles in the regulation of intracellular cyclic nucleotide amplitude, duration, and compartmentalization [3]. Alterations in PDE expression and activity are responsible for disruptions in cyclic nucleotide homeostasis, contributing to disease progression [3]. In this review, we give an overview of the role and therapeutic potential of PDE1 regulation in pathological cardiac remodeling and dysfunction. 

## 2. Pathological Cardiac Remodeling and Heart Failure

The heart is comprised of both cardiac myocytes and non-myocytes, including fibroblasts, endothelial cells, mast cells, and smooth muscle cells, as well as extracellular matrix [4]. In response to pathological conditions such as hypertension, neurohumoral activation, obesity, valvular heart disease, myocardial injury, and genetic mutations, the heart can undergo pathological remodeling, which features an increase in myocyte size, increased levels of myocyte death, and extracellular matrix protein deposition [5]. Cardiac hypertrophy is an adaptive response to increased workload and initially helps to maintain an almost normal function [1]. However, with prolonged stress, the heart can undergo irreversible decompensation, which is associated with a complex array of unfavorable events [1]. These pathological events include myocyte elongation and myocyte loss, which leads to chamber dilation and the thinning of the ventricular walls [1,6]. In addition, chronic stress stimulates cardiac fibroblast activation, characterized by a phenotypic changes to alpha-smooth muscle actin (α-SMA)-positive myofibroblasts [7]. Activated fibroblasts gain functions in cell proliferation, migration, and extracellular matrix (ECM) production, which leads to cardiac fibrosis, a hallmark of pathological cardiac remodeling [7,8]. Moreover, cardiac myocyte death is associated with various types of cardiac disease [9]. Apoptosis, necrosis, and autophagy are the three major types of cell death found in the heart, which induce cardiac arrhythmia, trigger cardiac remodeling, and ultimately lead to cardiac dysfunction [10]. Therefore, limiting cardiac myocyte loss by attenuating cell death has critical implications for the treatment of heart failure.

Numerous signaling pathways and molecular mechanisms have been implicated in cardiac hypertrophy [11]. For example, calcium (Ca^2+^)-dependent signaling molecules such as Ca^2+^/calmodulin-dependent serine/threonine phosphatase, calcineurin, and the Ca^2+^/calmodulin-dependent kinase II (CaMKII) play well-known roles in pathological cardiac remodeling [12]. Calcineurin has been implicated in mediating pathological hypertrophy in conjunction with the transcription factor nuclear factor of activated T cells (NFAT) [1,12]. Upon dephosphorylation by calcineurin in the cytosol, NFAT is imported into the nucleus and thus serves to induce a hypertrophic response [1]. The inhibition of calcineurin alleviates cardiac myocyte hypertrophy induced by Ang II or α-adrenergic agonist stimulation in vitro, and by pressure overload and isoproterenol (ISO) treatment in vivo [13]. The expression and phosphorylation of CaMKII can also be induced by an upregulation of intracellular Ca^2+^ levels in the cardiac myocyte [14]. In cardiac hypertrophy, CaMKII exerts its molecular effects by binding to and phosphorylating target proteins such as class II histone deacetylases (HDACs). Upon phosphorylation by CaMKII, several class II HDACs regulate the derepression of myocyte enhancer factor 2 (MEF2)-dependent genes through the nuclear export of HDACs, which contributes critically to hypertrophic signaling in the heart [15].

## 3. Cyclic Nucleotide Signaling and PDEs in the Heart

### 3.1. Cyclic 3′,5′ Adenosine Monophosphate (cAMP) Signaling

Cyclic AMP was first isolated and characterized by Sutherland and Rall over a half-century ago [16,17]. A large family of enzymes known as the adenylyl cyclases (ACs) catalyze the synthesis of cAMP from adenosine triphosphate (ATP) in response to a variety of extracellular signals such as hormones, growth factors, and neurotransmitters [18,19]. To date, 10 ACs, including nine membrane ACs and one soluble AC, have been identified [20,21]. Membrane ACs are regulated by G-protein coupled receptors (GPCRs), whereas soluble ACs are regulated by bicarbonate and Ca^2+^ [20,21,22]. This increase in cAMP leads to the activation of several downstream effector molecules including protein kinase A (PKA), an exchange factor directly activated by cAMP (EPAC), and cyclic nucleotide gated channels (CNGs) [18]. The development of Förster resonance energy transfer (FRET)-based techniques that enable the measurement of cAMP concentrations at the single cell level have demonstrated that cAMP levels are not uniform throughout the entire cell, which may indicate that it regulates different biological functions [23,24].

In the cardiac myocyte, catecholamines stimulate the β-adrenergic receptors (β-ARs), leading to cAMP-mediated activation of PKA signaling, which then is able to phosphorylate L-type Ca^2+^ channels (LTCC) and ryanodine receptors (RYRs) to induce intracellular Ca^2+^ increases, thus stimulating myocyte contractility [25,26,27,28]. Furthermore, the PKA-mediated phosphorylation of phospholamban (PLB) leads to enhanced Ca^2+^ re-uptake through the sarcoplasmic/endoplasmic reticulum calcium ATPase 2 (SERCA2) in the sarcoplasmic reticulum (SR) adjacent to the T-tubule, leading to faster myofilament relaxation [29]. β-AR mediated cAMP signaling can be either detrimental or beneficial to the heart. Acute stimulation of β1-AR/cAMP has beneficial effects on cardiac contractile function, whereas chronic stimulation results in myocyte hypertrophy, apoptosis, and cardiac fibrosis, finally leading to heart failure [30]. Interestingly, it has been found that the activation of cAMP/PKA signaling by stimulating the adenosine A_2_ receptor (A_2_R) is cardiac-protective: for example, A_2_R activation alleviated transverse aortic constriction (TAC)-induced cardiac dysfunction [31]. These studies indicate that different cAMP signaling modules regulate distinct and even opposing effects in cardiac myocytes.

### 3.2. Cyclic 3′,5′ Guanosine Monophosphate (cGMP) Signaling

Cyclic GMP is generated from GTP by two families of guanylyl cyclases (GCs), with seven particulate GCs (pGCs) activated by natriuretic peptides (NPs) and three soluble GCs (sGCs) activated by nitric oxide (NO) or carbon monoxide (CO) [32,33]. cGMP regulates three major types of effector molecules: cGMP dependent protein kinases (PKG), cGMP-regulated PDEs, and cGMP-gated cation channels [34]. Similar to cAMP, cGMP is highly compartmentalized in the cell, and elicits distinct downstream biological effects [35,36]. There appear to be multiple protective cGMP signaling modules in the heart [37,38,39,40]. For example, a study by Frantz and colleagues found that mice with a cardiac myocyte-specific PKGI knockout (KO) developed significant cardiac dysfunction and cardiomyopathy in both Ang II- and TAC-induced cardiac hypertrophic models, with decreased expression of SERCA2 and PLB and altered Ca^2+^ homeostasis [37]. PDE5 inhibition has been shown to attenuate cardiac hypertrophy by targeting NO-sGC-derived cGMP [38,40,41,42,43]. PDE9 inhibition also protects against cardiac hypertrophy by targeting NP-pGC-derived cGMP [44]. Similarly, we have also found that PDE1A inhibition attenuates cardiac myocyte hypertrophy in a cGMP-dependent manner [45]. 

### 3.3. Different PDE Isozymes in the Heart

The degradation of cyclic nucleotides is catalyzed by PDEs. The PDE superfamily is comprised of 11 structurally related but functionally distinct gene families, PDE1–PDE11, with differences in their cellular functions, structures, catalytic properties, and mechanisms of regulation [3]. All PDEs share a conserved carboxyl-terminal catalytic core of approximately 270 amino acids, with a shared sequence identity of 25–50% between family members [46]. However, the regulatory characteristics of PDEs are determined by their unique amino-terminal regions, which contain elements known to be involved in enzyme phosphorylation, dimerization, auto-inhibition, ligand binding, and protein-protein interaction. These regions thus allow distinct localization and heterogeneity in cyclic nucleotide signaling and enable diverse functions of various PDEs [3,46,47]. 

Research has clearly established that the degradation of cyclic nucleotides by PDEs is regulated differently under physiological and pathological conditions. In particular, the expression and activity of several PDEs are altered in various types of cardiovascular disease [40,44,45,48,49]. Up to now, at least seven PDE family members have been found in the heart, including PDE1–PDE9 [44,48,50,51,52,53,54]. Among these, PDE4 and PDE8 specifically hydrolyze cAMP, PDE5 and PDE9 specifically hydrolyze cGMP, while PDE1, 2, and 3 can hydrolyze both cAMP and cGMP [3]. Thus, a thorough understanding of the roles of specific PDE isoforms in cardiac pathology and physiology is critical. This review mainly focuses on the roles of two isoforms of PDE1 in the heart (PDE1A and PDE1C).

## 4. PDE1 and Pathological Cardiac Remodeling and Dysfunction

### 4.1. PDE1

The PDE1 family was one of the first classes of PDEs to be identified [55]. All PDE1 family members are regulated by the binding of Ca^2+^/CaM, which is the reason that PDE1 is also referred to as Ca^2+^/CaM-stimulated PDE [56]. Thus, the Ca^2+^-dependent activation of PDE1 isozymes plays a critical role in the crosstalk between Ca^2+^ and cyclic nucleotide signaling [57]. The PDE1 family members are encoded by three distinct genes, *PDE1A*, *1B,* and *1C*, and alternative splicing of these genes gives rise to a number of functionally distinct isozymes, which allows for differential cell/subcellular expression and Ca^2+^ sensitivity, leading to the finely tuned regulation of cyclic nucleotide signaling [45]. Like other PDEs, PDE1 variants consist of a conserved C-terminal catalytic domain with diverse N-terminal regulatory domains. The N-terminus of all PDE1s contains two CaM binding domains with an inhibitory sequence in between them. It has been suggested that the binding of Ca^2+^/CaM therefore relieves the inhibition of PDE1 activity [58]. As a consequence, the catalytic activity of PDE1 can be stimulated more than 10-fold upon the binding of Ca^2+^/CaM [56,59]. Each PDE1 isoform is able to catalyze the hydrolysis of both cAMP and cGMP, but with different substrate affinities. PDE1As hydrolyze cGMP (*K*m ≈ 5 μM) with greater affinity than cAMP (*K*m ≈ 112 μM) in vitro [59]. PDE1B enzymes also prefer cGMP (*K*m ≈ 2.4 μM) to cAMP (*K*m ≈ 24 μM) [49]. However, PDE1Cs hydrolyze cAMP and cGMP with a similarly high affinity (*K*m ≈ 1 μM) [49,56].

Given the well-established role of Ca^2+^ signaling in pathological cardiac remodeling and the potential role of PDE1 as a mediator for Ca^2+^ to antagonize cyclic nucleotide signaling, understanding the role of PDE1 in cardiac diseases is of great interest. PDE1 expression is highly regulated with differential isozyme localization to specific tissues and cell types [50,58,60,61]. PDE1A expression is relatively low in the normal heart but is upregulated in the diseased heart, which is consistent among humans, rats, and mice [62]. PDE1C expression varies with species: it is high in human, modest in mouse, and low in rat normal heart [45,62,63]. PDE1B expression is barely detectable in the heart [62,63]. In human myocardium, PDE1C has been shown to be localized along the M- and Z-lines of cardiac myocytes in a striated pattern [62]. Taken together, these observations indicate that there are substantial species-specific differences in PDE1 expression in the heart. With the development of PDE1-selective inhibitors and the genetically engineered PDE1 KO mice, the roles of PDE1 in cardiac cells and cardiac disease models have been recently explored in vitro and in vivo. Here we review the expression and regulation of different PDE1 isoforms in pathological cardiac remodeling and dysfunction.

### 4.2. Role of PDE1A in Cardiac Myocyte Hypertrophy and Fibroblast Activation

PDE1A expression has been found to be significantly upregulated in diseased hearts of various etiologies, such as in mouse hearts with dysfunction induced by chronic ISO infusion, myocardial infarction (MI), and TAC, in rat heart treated with chronic Ang II infusion, as well as in human failing hearts with both dilated and ischemic cardiomyopathy [36,45]. PDE1A induction in diseased hearts appears to occur in both cardiac myocytes as well as in activated cardiac fibroblasts in fibrotic areas [36]. PDE1A upregulation has been also observed in cultured neonatal rat ventricular myocytes (NRVMs) and adult rat ventricular myocytes (ARVMs) given hypertrophic stimuli such as ISO or Ang II [45]. To determine the specific contribution of PDE1A to the regulation of cardiac hypertrophy, Miller et al. utilized the pan PDE1-selective inhibitor IC86340 and PDE1A-specific shRNA to block PDE1A function in rat cardiac myocytes where PDE1C expression is limited [45]. They found that PDE1 inhibition by IC86340 and PDE1A downregulation by shRNA prevent the hypertrophic response induced by phenylephrine (PE) treatment in both NRVMs and ARVMs. PE causes a reduction in cGMP levels in cardiac myocytes, which can be abolished by the PDE1 inhibitor IC86340 or PDE1A shRNA [45]. This suggests that PDE1A plays a critical role in the PE-induced suppression of cGMP signaling in cardiac myocytes, likely through the Ca^2+^-dependent activation of PDE1A. Consistently, PDE1A suppression of cardiac hypertrophy also occurs in a PKG-dependent manner [45]. These studies indicate that the inhibition of PDE1A alleviates cardiac myocyte hypertrophy by blocking a PE-induced decrease in intracellular cGMP and PKG activity [45]. However, the detailed underlying molecular mechanism by which PDE1A-mediated cGMP/PKG signaling regulates cardiac myocyte hypertrophy remains unknown. PDE5A inhibition also suppressed cardiac myocyte hypertrophy in a cGMP/PKG-dependent manner [38]. However, the mechanisms by which PDE1A and PDE5A mediate the regulation of cardiac myocyte hypertrophy appear to be different. This is supported by the fact that treatment with the PDE5 inhibitor sildenafil together with the PDE1 inhibitor IC86340 or PDE1A shRNA elicited additive effects on antagonizing myocyte hypertrophy, suggesting that two different pathways are involved [45].

In addition to cardiac myocytes, PDE1A is also significantly induced in activated cardiac fibroblasts (α-SMA positive myofibroblasts) within fibrotic areas of rodent and human failing hearts [36]. In vitro, PDE1A can be upregulated in rat cardiac fibroblasts by treatment with pro-fibrotic stimuli such as Ang II and transforming growth factor β (TGF-β) [36]. In addition, the PDE1 family contributes 70% of total cGMP-hydrolyzing PDE activity under Ang II stimulation, suggesting a major role for PDE1 in Ang II-activated fibroblasts [36]. The role of PDE1A in cardiac fibroblast activation (phenotype changing to myofibroblasts) was investigated in primary cultured rat cardiac fibroblasts [36]. Both IC86340 and PDE1A shRNA treatment reduced Ang II- or TGF-β-induced myofibroblast activation and ECM synthesis [36]. PDE1A-mediated myofibroblast transformation and ECM synthesis are dependent on the suppression of both cAMP/Epac1/Rap1 signaling as well as cGMP/PKG signaling [36], which is in line with the dual substrate specificity of PDE1A. FRET-based approaches further showed that IC86340 induces a rapid but transient elevation of cGMP, and preferentially stimulated nuclear and perinuclear cAMP production in activated fibroblasts [36]. The roles of PDE1A-mediated regulation of cAMP/Epac1 and cGMP/PKG in cardiac fibroblasts are consistent with previous findings that the activation of Epac1 [64,65,66] or cGMP/PKG signaling [38,67,68] exerts anti-fibrotic effect in cardiac fibroblasts.

Recently, Wang et al. generated two different lines of global PDE1A null mice by using the transcription activator-like effector nucleases (TALEN) approach, one containing a frame deletion of 15bp within the catalytic active site of mPDE1A and the other exhibiting a frame shift insertion, for the purpose of studying polycystic kidney disease (ADPKD) [69]. It has been shown that both null lines of mice developed a mild renal cystic disease phenotype on a wild-type background and accelerated the development of phenotype in an ADPKD mouse model (a *pkd2* gene mutant background) at ages ≤16 weeks [69]. Additionally, these PDE1A null mice had lower aortic blood pressure and increased left ventricular ejection fraction [69]. However, whether PDE1A deficiency affects stress-induced pathological cardiac remodeling and dysfunction is still not clear.

### 4.3. Role of PDE1C in Pathological Cardiac Remodeling and Dysfunction

PDE1C represents one of the major PDE activities in the normal human heart [62]. Its role in cardiac remodeling and dysfunction was recently evaluated by Knight et al. using a global PDE1C knockout (PDE1C-KO) mouse strain. In their study, it was found that PDE1C expression is upregulated in both mouse and human failing hearts [49], which is consistent with a recent RNA-sequencing study of human failing heart samples reporting an increase of PDE1C expression in both ischemic heart disease and dilated cardiomyopathy [49,70]. Unlike PDE1A, PDE1C is only expressed in cardiac myocytes and is undetectable in cardiac fibroblasts or myofibroblasts [49]. Interestingly, TAC-induced cardiac remodeling and dysfunction observed in PDE1C wild-type (PDE1C-WT) mice was significantly alleviated in PDE1C-KO mice, as indicated by reduced chamber dilation, myocardial hypertrophy, cardiac myocyte apoptosis, and interstitial fibrosis, as well as attenuated loss of fractional shortening and ejection fraction [49]. This indicates a detrimental role for PDE1C in the development of heart failure induced by chronic pressure overload.

In isolated cardiac cells, PDE1C deficiency abolished Ang II-, PE-, or ISO-induced cardiac myocyte hypertrophy [49]. Ang II- and ISO-induced cell death were also blocked in PDE1C-KO myocytes. In addition, IC86340 attenuated cell death and apoptosis in WT myocytes but had no further effect in PDE1C-KO myocytes, indicating that the protective effect of IC86340 on myocyte death is primarily through PDE1C [49]. The anti-hypertrophic and anti-apoptotic effects of PDE1C deficiency and/or inhibition were mediated in a cAMP/PKA-dependent manner, and the PI3K/AKT signaling pathway appears to be important for this protective effect [49]. However, the detailed molecular mechanisms by which PDE1C regulates cAMP-mediated cardiac-protective signaling pathways deserve to be further investigated. PDE1C expression was barely detectable in WT cardiac fibroblasts or WT myofibroblasts (stimulated with TGF-β) [49]. However, TAC-induced cardiac interstitial fibrosis was attenuated in PDE1C-KO hearts [49]. This raises the hypothesis that myocyte PDE1C regulates fibroblast function through a paracrine-dependent mechanism. Indeed, conditioned medium collected from PDE1C-KO but not PDE1C-WT cardiomyocytes was found to attenuate TGF-β-induced fibroblast activation, suggesting that PDE1C inhibition or deficiency inhibits fibroblast activation through secreted factor(s) from myocytes [49]. However, the secreted factor(s) that mediate this crosstalk between cardiac myocytes and fibroblasts remain unknown.

### 4.4. PDE1 and Other PDEs: Similarities, Differences, and Potential Interactions

Although PDE1A is able to regulate cGMP levels and cGMP-dependent signaling in cardiac myocytes [45], the specific source(s) of cGMP modulated by PDE1A in cardiac myocytes remains unknown. In vascular SMCs, PDE1A appears to be able to regulate cGMP derived from atrial natriuretic peptide (ANP) stimulation [71]. Several other PDEs have previously been shown to regulate cGMP signaling in cardiomyocytes. For example, PDE5A likely regulates NO-derived cGMP [38] and plays a critical role in mediating various types of cardiac diseases including ischemia/reperfusion (I/R) injury [72,73], doxorubicin cardiotoxicity [74], ischemic and diabetic cardiomyopathy [75], and cardiac hypertrophy [38,76]. PDE5 is expressed at low levels and localized to sarcomeric Z-bands within cardiac myocytes [53]. A recent study found that PDE9, another cGMP-specific PDE, upregulated expression in both mouse and human hypertrophic hearts [44]. The inhibition and genetic deletion of PDE9 was shown to protect the heart against TAC-induced cardiac remodeling and pre-established heart disease [44]. PDE9 couples to NP-derived cGMP [44]. Moreover, PDE2, which preferentially localizes in the membrane fraction of cardiac myocytes, is also critical for cGMP catabolism in cardiac myocytes in response to particulate GC activation [35]. Evidence to date suggests that PDE1C primarily regulates cAMP signaling in cardiac myocytes [49]. PDE1C inhibition/deficiency protects cardiac myocyte from death in a PKA-dependent manner [49]. Also, PDE1C is responsible for the Ang II-mediated suppression of myocyte cAMP levels [49]. These observations suggest that PDE1C antagonizes a protective cAMP signaling pathway in cardiac myocytes. Indeed, our unpublished observations suggest that PDE1C negatively regulates cardiac protective A_2_R-cAMP signaling. In contrast, previous studies have shown that PDE3A inhibition potentiates a detrimental cAMP signaling pathway that promotes cardiac myocyte death [48,77,78,79]. However, PDE4 inhibition has a limited effect on cardiac myocyte death/survival despite the drastic elevation of cAMP upon PDE4 inhibition [48].

Taken together, these findings suggest that in cardiac myocytes there are multiple different, even functionally opposing cyclic nucleotide signaling pathways that are modulated by distinct PDE isozymes. The functional diversity of individual PDEs could be achieved through multiple mechanisms: the association of PDEs with discrete “pools” of cyclic nucleotides, targeted to spatially separated compartments, and complexed with distinct sets of signaling molecules. In addition, the variable enzymatic and regulatory properties make individual PDEs function in different biological conditions, such as basal vs. stimulated, physiological vs. pathological cellular environments, etc. PDE1-mediated functions are associated with intracellular Ca^2+^ levels because PDE1 needs Ca^2+^ to be activated. For example, previous studies have shown that PDE1A is responsible for PE-induced cGMP reduction, while PDE1C is important for Ang II-induced cAMP reduction in cardiac myocytes [45,49]. Moreover, a recent study using FRET approaches further supported the role of Ca^2+^ in the PDE1-mediated regulated cAMP response in cardiac myocytes [80]. Specifically, it has been shown that PDE1 inhibition is able to elicit significant cAMP elevation under stimulation by forskolin but not ISO, under a paced but not basal state, or mimicked by pretreating resting cells with Ca^2+^ elevating agents [80]. It will be of great interest to understand the integratory roles of these different cyclic nucleotide signaling pathways modulated by distinct PDEs in regulating cardiac myocyte functions. Targeting multiple PDEs in combination may represent a compelling strategy. Indeed, a number of recent studies have shown synergistic effects on cyclic nucleotide level and subsequent biological functions resulting from the inhibition of combinations of PDEs [81,82].

## 5. Therapeutic Potential of PDE1 Inhibition in Pathological Cardiac Remodeling

To evaluate the pharmacological effects of PDE1 inhibition in pathological cardiac remodeling in vivo, the systemic application of IC86340 was evaluated in an ISO-induced mouse hypertrophy and fibrosis model [36,45]. It was found that mice receiving daily IC86340 treatment at the doses of 3 and 6 mg/kg per day elicited a small (10 to 20 mmHg) reduction of blood pressure (BP) with no significant changes in heart rate. The effect on BP reduction is consistent with that of other PDE1 inhibitors such as Lu AF41228/Lu AF58027 and vinpocetine, which have been shown to cause vasodilation and/or lower BP in rodents [71,83]. The effects of PDE1 inhibitors on blood pressure regulation are likely mediated exclusively by PDE1A, but not PDE1C, as PDE1A is expressed in the medial vascular smooth muscle cells (SMCs) responsible for vascular contractile function [84,85,86]. The role of PDE1A in the regulation of blood pressure has been more directly supported by the recent findings from two lines of PDE1A null mice that showed reduced aortic blood pressure [69]. Additionally, the association of PDE1A single nucleotide polymorphisms (SNPs) with diastolic blood pressure has been revealed by human genetic studies [87,88]. In addition to the effect of IC86340 on BP, there is no correlation between BP and ISO-induced remodeling [89]. In the mouse heart, IC86340 treatment (3 mg/kg per day) attenuated ISO infusion-mediated increases in cardiac hypertrophy, as assessed by increased heart size, heart weight/body weight ratio, and heart weight/tibia length ratio [45]. In addition, enlarged cardiac myocyte cross-sectional area and elevated ANP gene expression were also significantly reduced by IC86340 treatment [45]. Moreover, cardiac fibrosis, as indicated by α-SMC-positive myofibroblasts and collagen deposition, were significantly reduced by IC86340 [36]. These results support a critical role for PDE1 activation in ISO-induced cardiac hypertrophy in vivo. The effects of IC86340 or other PDE1-selective inhibitors deserve to be evaluated in different models of cardiovascular disease in the future. 

Vinpocetine is also widely used as a PDE1 inhibitor. It is a derivative of the alkaloid vincamine and has, for several decades now, been used clinically in many countries for the treatment of cerebrovascular disorders such as stroke and dementia [90,91,92]. In addition to being a PDE1 inhibitor, vinpocetine can act as a blocker for voltage-dependent Na^+^ channels as well as act as an inhibitor for IkB kinase (IKK), serving as a critical mediator of inflammatory signaling [93]. The excellent safety profile of vinpocetine has attracted significant interest for exploring the potential therapeutic effects of vinpocetine treatment in various other disease models. Indeed, emerging experimental evidence has shown the beneficial effects of vinpocetine in various inflammatory and cardiovascular disease models. For example, vinpocetine treatment partially restored the sensitivity of the vasculature to nitroglycerin (NTG) in a rat model of NTG tolerance [71]. In addition, vinpocetine significantly attenuated mouse carotid artery wall thickening and neointimal formation induced by ligation injury, and markedly suppressed the spontaneous remodeling of human saphenous vein explants in an ex vivo culture model [94]. High-fat diet-induced atherosclerosis in ApoE-deficient mice was also significantly suppressed by vinpocetine [95,96]. Moreover, the inhibition of PDE1 by vinpocetine has been suggested to play a protective role in cardiac hypertrophy and fibrosis [97]. It has been found that vinpocetine significantly alleviates chronic Ang II-induced cardiac hypertrophy and fibrosis in vivo [97]. Consistently, in isolated cardiac myocytes, vinpocetine attenuates Ang II-induced myocyte hypertrophy in a dose-dependent manner [97]. In addition, vinpocetine blocks TGF-β-induced fibroblast activation [97]. Interestingly, when PDE1 activity is blocked by IC86340, vinpocetine exerts no additional effects on cardiac myocyte hypertrophy and fibroblast activation, likely demonstrating that its protective effects are effective largely through PDE1 inhibition [97]. Taken together, these data indicate that vinpocetine is a drug with multiple pharmacological targets and multiple therapeutic effects [93]. In addition to its direct protective effects on cardiac cells, other biological effects of vinpocetine may also act together in an indirect beneficial manner for preventing cardiac diseases, including vasodilation, anti-inflammation, and anti-vascular occlusive remodeling [93,94,98,99,100].

## 6. Conclusions and Perspective

**Figure 1:**
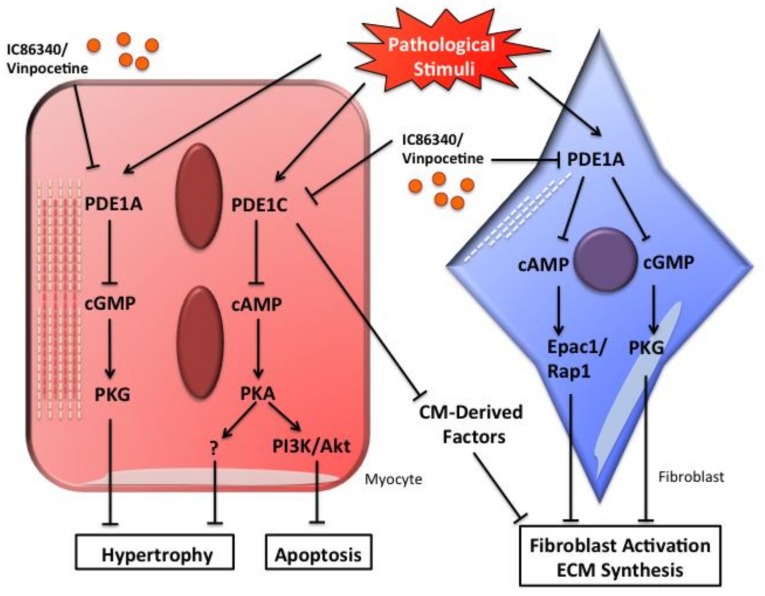
Schematic diagram showing the potential role of PDE1A and PDE1C in regulating pathological cardiac remodeling including cardiac hypertrophy and fibrosis. In cardiac myocytes, PDE1A-dependent regulation of cGMP/PKG signaling alleviates myocyte hypertrophy, PDE1C-dependent regulation of cAMP/PKA signaling suppresses both myocyte hypertrophy and apoptosis. In cardiac fibroblasts, PDE1A-dependent dual regulation of cAMP-Epac1-Rap1 and cGMP-PKG signaling decreases fibroblast activation and ECM synthesis, whereas PDE1C may regulate cardiac fibrosis through paracrine signaling between cardiac myocytes and fibroblasts.

Experimental evidence has suggested that both PDE1A and PDE1C are important in regulating cardiac structural remodeling and function (Figure 1). However, their regulation, function, and mechanistic actions in the heart appear to be distinct. PDE1A is expressed and functions in both cardiac myocytes and fibroblasts, particularly in response to stimulation with hypertrophic and fibrotic stimuli [36,45]. However, PDE1C is expressed in cardiac myocytes but not fibroblasts [49]. PDE1A and PDE1C both regulate cardiac myocyte hypertrophy induced by pathological stimuli such as Ang II, but via distinct signaling pathways: PDE1A acts through cGMP/PKG while PDE1C acts through cAMP/PKA [45,49]. The differential functions and mechanisms of PDE1A and PDE1C in cardiac hypertrophy stimulated with other growth factors or particularly in response to physiological hypertrophy also deserve to be further investigated. Interestingly, the protective effect of pan PDE1 inhibition on cardiac myocyte death is primarily dependent on PDE1C inhibition [49]. PDE1C deficiency but not PDE1A deficiency blocked myocyte death induced by different death stimuli (our unpublished observations). These results suggest that PDE1C but not PDE1A plays a major role in regulating cardiac myocyte death, at least in isolated cardiac myocytes in vitro. In cardiac fibroblasts, PDE1A directly regulates fibroblast activation and ECM production [36]. However, PDE1C appears to regulate fibroblast function through the myocyte-PDE1C-mediated regulation of unknown secreted factor(s) via a paracrine mechanism [49]. Most knowledge of PDE1A in cardiac myocytes and fibroblasts is derived from experimental observations in cultured primary cells [36,45,49]. Future validation in animal models in vivo is necessary. Taken together, PDE1A and PDE1C regulate distinct cyclic nucleotide signaling pathways and play different roles in the heart. Future development of cardiac cell-specific PDE1A or 1C knockout mice, or genetically modified mice with selectively altered PDE1A or PDE1C, should be helpful in elucidating a more detailed characterization of the role of PDE1 isoforms in cardiac remodeling and dysfunction.

Although both PDE1A and PDE1C exhibit direct biological effects in cardiac myocytes and/or fibroblasts, the two major cell types in the heart, the expression of PDE1 isozymes in other cardiac cell types or in other organs may also indirectly influence cardiac structural remodeling and function [36,45,49]. These tissues/organs include but are not limited to the vasculature, brain, kidney, and lung. PDE1A and 1C have very different expression profiles in many tissues of the human body [63]. PDE1A null mice have been shown to have a mild renal cystic disease and a urine concentrating defect [69]. Moreover, a recent study defined a significant level of expression of PDE1A in sinoatrial nodal tissue, which may be important in regulating sinoatrial nodal pacemaker function [101]. Therefore, when studying cardiac function using global PDE1A knockout mice or systemically applying pan PDE1 inhibitors, the potential effects on heart rate, contractility, BP reduction, and kidney function should be taken into consideration.

To date, all PDE1 inhibitors are pan inhibitors, lacking the ability to distinguish between different PDE1 isozymes. The future development of PDE1 isozyme-selective inhibitors is important for achieving specific pharmacological effects. Taken together, basic research studies in isolated cardiac cells and in experimental cardiac disease models provide strong evidence for roles of PDE1A and PDE1C in pathological cardiac remodeling and dysfunction, suggesting that PDE1A and PDE1C might represent potential novel and promising therapeutic targets.
